# Cultivar architecture modulates spore dispersal by rain splash: A new perspective to reduce disease progression in cultivar mixtures

**DOI:** 10.1371/journal.pone.0187788

**Published:** 2017-11-15

**Authors:** Tiphaine Vidal, Pauline Lusley, Marc Leconte, Claude de Vallavieille-Pope, Laurent Huber, Sébastien Saint-Jean

**Affiliations:** 1 UMR ECOSYS INRA, AgroParisTech, Université Paris-Saclay 78850 Thiverval-Grignon, France; 2 UMR BIOGER INRA, AgroParisTech, Université Paris-Saclay 78850 Thiverval-Grignon, France; Fujian Agriculture and Forestry University, CHINA

## Abstract

Cultivar mixtures can be used to improve the sustainability of disease management within farming systems by growing cultivars that differ in their disease resistance level in the same field. The impact of canopy aerial architecture on rain-splash dispersal could amplify disease reduction within mixtures. We designed a controlled conditions experiment to study single splash-dispersal events and their consequences for disease. We quantified this impact through the spore interception capacities of the component cultivars of a mixture. Two wheat cultivars, differing in their aerial architecture (mainly leaf area density) and resistance to Septoria tritici blotch, were used to constitute pure stands and mixtures with 75% of resistant plants that accounted for 80% of the canopy leaf area. Canopies composed of 3 rows of plants were exposed to standardized spore fluxes produced by splashing calibrated rain drops on a linear source of inoculum. Disease propagation was measured through spore fluxes and several disease indicators. Leaf susceptibility was higher for upper than for lower leaves. Dense canopies intercepted more spores and mainly limited horizontal spore transfer to the first two rows. The presence of the resistant and dense cultivar made the mixed canopy denser than the susceptible pure stand. No disease symptoms were observed on susceptible plants of the second and third rows in the cultivar mixture, suggesting that the number of spores intercepted by these plants was too low to cause disease symptoms. Both lesion area and disease conditional severity were significantly reduced on susceptible plants within mixtures on the first row beside the inoculum source. Those reductions on one single-splash dispersal event, should be amplified after several cycle over the full epidemic season. Control of splash-dispersed diseases within mixtures could therefore be improved by a careful choice of cultivars taking into consideration both resistance and architecture.

## Introduction

Agriculture is currently facing the challenge of feeding a growing world population while maintaining environmental sustainability [1]. Plant diseases are responsible for high losses of crop production [2] and therefore require efficient management. However, commonly used tools such as fungicides and resistant cultivars have limitations. Fungicides are responsible for environmental problems and can lose their efficiency due to fast pathogen adaptation [3]. Similarly, using almost exclusively a very limited number of highly resistant cultivars can lead to resistance break-down in some cases [4]. In other cases, only partially resistant cultivars are available, requiring complementary management techniques [5].

Alternative disease management can be achieved by taking advantage of agrobiodiversity [6]. For example growing together a susceptible and a resistant cultivar within the same field can reduce disease on susceptible plants compared to pure stand [7]. This cultural practice, cultivar mixture, can be used as a complementary tool to improve the sustainability of crop production, with only minor modifications to mechanized farming systems. Despite their relatively low adoption in the field, cultivar mixtures are an interesting option of agroecological management of crop diseases. Disease reduction on susceptible plants within cultivar mixtures can be explained by several mechanisms [6]. Major ones are related to modifications of pathogen spore dispersal within mixtures: (i) density effect [8] results from increased distances between susceptible plants compared to pure stands, which reduce spore transfer from one susceptible plant to another, (ii) barrier effect [9] results from spore interception by resistant plants, which prevents part of the inoculum from reaching susceptible plants. The spore interception capacities of plants, which can be assessed in pure stands, are therefore critical in cultivar mixtures.

Cultivar mixtures can provide control of both wind-dispersed [7, 10] and soil-borne [11] fungal diseases, thus offering a possibility to reduce use of fumigant and fungicide. However, mixture effects are variable in the case of splash-dispersed diseases [12, 13] such as Septoria leaf blotch of wheat (STB), a major wheat disease [14, 15] caused by *Zymoseptoria tritici* [16], which is mainly splash-dispersed during the epidemic phase. Wind and splash dispersal differ mainly in terms of dispersal scale: wind-dispersed spores can be transported over several kilometers while rain-dispersed spores rarely travel further than one meter from their source plant [17, 18]. Wind dispersal generally leads to higher protective effects within mixtures mainly because allo-contamination is lower [19].

Several authors have observed an effect of aerial canopy architecture on splash dispersal [9, 20, 21]. Canopy architecture results from architectural characteristics of individual plants [22]. Plant architectural traits such as number of tillers, leaf dimensions and vertical distance between leaves thus determine canopy properties such as leaf area index (LAI) and density (LAD) as well as porosity. These properties determine the medium of spore dispersal and can have an impact on disease [23, 24]. Aerial architecture of plants composing a cultivar mixture canopy could therefore have an impact on spore dispersal and related mechanisms of disease reduction. Taking plant architecture into account in the process of mixture design could therefore be a way of reducing the propagation of splash-dispersed diseases [25]. However, little quantitative information is available on the contribution of plant architecture to the reduction of splash-dispersed disease propagation within cultivar mixtures.

In order to provide disease control, cultivar mixtures must contain plants differing in their level of resistance to disease. In a mixed canopy, (i) plants produce spores according to their level of susceptibility to the pathogen. During rain events, (ii) these spores are dispersed by rain-splash throughout the canopy and generate spore fluxes. Spores are transfered according to canopy architecture and are (iii) eventually intercepted by both susceptible and resistant plants, leading to barrier and density effects [8]. After a latency period, (iv) spore interception leads to disease symptoms according to host resistance. The sporulating lesions constitute new sources of spores for the next dispersal event (v). Mixture effects are thus amplified cycle after cycle (vi). In field conditions, architecture and host resistance are strongly entangled in these processes and their study requires specific experimental techniques.

Our objective was to assess the impact of architecture on spore interception (iii) in the case of a cultivar mixture. In order to focus on spore interception, we designed a specific experiment where the production of spore fluxes (i and ii) is standardized. This artificial spore flux was independent of host resistance and was the same for all treatments. Single dispersal events (*i.e*. one disease cycle, from spore interception to new sporulating lesions) were studied under controlled conditions for both pure stands and mixed wheat canopies.

The first step of our work was to assess the difference of spore interception properties between two cultivars differing by their architecture, in pure stand. The second step was to assess the impact of this difference of architecture in the case of a cultivar mixture. The effect of architecture in the mixture was quantified by comparing disease intensity on susceptible plants (same host resistance) grown in pure stand and in mixture. This made it possible to disentangle the effect of architecture and host resistance that determine disease intensity resulting from spore dispersal (iv) and to better understand the contribution of cultivar architecture in the control of splash-dispersed diseases within cultivar mixtures (iii).

## Materials and methods

Studying mechanisms linked to dispersal in natural epidemics is not a trivial matter. Septoria tritici blotch (STB) is a particularly challenging case due to its poly-cyclicity and the usually widespread distribution of inoculum throughout fields. Moreover, STB has a long latency period of about 3 weeks between a rain-dispersal event and symptom appearance. As new dispersal events can occur during latency, it is often difficult to relate symptoms to a particular rain event. In order to better understand factors that determine the spore interception properties of different cultivars, we studied single dispersal events under controlled conditions, from spore interception to the appearance and severity of disease symptoms.

We used healthy adult plants grown together in a canopy. Leaf susceptibility of cultivars was assessed in a separate experiment, with non-limiting inoculum. Standardized spore fluxes were generated for two types of simulated rain in order to study canopy interception properties. Incoming spore fluxes had the same characteristics (location, number of spores and pathogen strain) for all canopies and were independent of both canopy architecture and cultivar resistance to disease. Splash dispersal patterns observed within canopies thus depended only on the architecture of each canopy. Spore fluxes were measured within canopies and in the absence of plants. Finally, symptoms resulting from dispersal events were measured in detail. Spatial organization of plant tissues was assessed using 3D numeric reconstruction of wheat canopies.

### Plants and canopy architecture

Two bread wheat cultivars with contrasting resistance levels (Sogood: susceptible 4/9 and Maxwell: partially resistant 7/9 score, on a 0-9 scale, 0 being the most susceptible case) were grown under controlled greenhouse conditions. Seeds were sown in individual pots. Seedlings were then vernalized (8 weeks at 6°C) and transplanted in line to form rows of 1 m length, in order to mimic field row sowing. Each row contained 40 plants, either of a single cultivar (pure stands) or of two cultivars (mixtures). In the case of cultivar mixtures, one susceptible plant was transplanted for every 3 resistant plants, corresponding to a proportion of 75% of partially resistant plants. These proportions are efficient in reducing disease spread in the field [13]. At flowering (growth stage 60 [26]), rows were arranged into canopies. At this stage, stem extension and leaf growth were both fully completed: wheat architecture was therefore considered as fixed. Each plant had on average 3 fully developed tillers (2.92 for Maxwell and 3.00 for Sogood). Canopies of about 0.5 m^2^ were constituted with 3 rows separated by inter-rows of 17 cm, corresponding to a density of 235 plants per m^2^, which is comparable with common field configurations for wheat crops. Each canopy was submitted to rain-splash inoculum a single time. Each repetition of the experiment included the application of two rain types on each of the three treatments (resistant pure stand, susceptible pure stand and cultivar mixture). Two repetitions were carried out.

**Fig 1:**
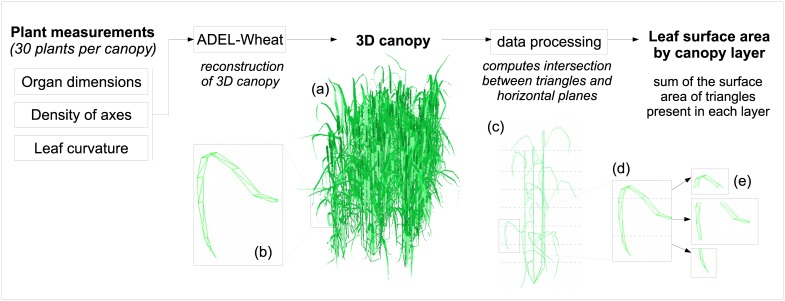
Assessing vertical distribution of leaf area using 3D canopies. (a) Realistic 3D canopies at flowering stage are generated from plant measurements, using ADEL-Wheat, a 3D architectural model of wheat development [27] (b) Detail of an individual leaf: each organ of the canopy is constituted of triangles, the output of the model is a list of triangle coordinates (c) Detail of an individual plant. Dashed lines represent horizontal planes delimiting canopy layers. (d) Detail of an individual leaf divided into horizontal canopy layers (e) Output of the data processing code is list of triangles for each layer, from which leaf surface area is computed, for each canopy layer.

Canopy architecture was important in our experiment as it constituted the dispersal medium of spores. In order to deduce the spatial organization of plant tissues from individual plant architectural traits, we used 3D reconstruction of wheat canopies based on detailed plant measurements (Fig 1). Leaf curvature was measured using a 3D digitizer (Polhemus, USA), leaf lamina dimensions as well as leaf insertion height were measured on 10 plants per row, in each canopy. Collected information was used to reconstruct static virtual canopies (hereafter called “mock-ups”) using an existing 3D architectural model of wheat development [27] (available from the OpenAlea plant-modeling platform [28]). This type of model was designed to provide a dynamic simulation of wheat canopy architectures from detailed descriptions of plant characteristics provided for a limited number of measuring dates. In our case, the model was used to enable the *in silico* construction of realistic virtual canopies at flowering stage based on experimental plant measurements. We computed the distribution of leaf surfaces by leaf rank as a function of canopy height in order to quantify the position of plant tissues in relation to spore fluxes. This variable was estimated by dividing 3D canopies into horizontal layers (Fig 1) using a dedicated data processing code (C++). Leaf Area Index (LAI: leaf surface area per area of ground) was assessed by measuring total leaf area and divided by ground surface area. Leaf Area Density (LAD: leaf surface area per volume of canopy) was obtained as the ratio of LAI and canopy height.

### Fungal pathogen inoculum and wheat cultivar susceptibility

Wild type isolates of *Z. tritici* were collected in the field, at Versailles near Paris (France), on wheat plants of the susceptible cultivar (Sogood) and grown on Petri dishes during 5 days at 18°C. Spore suspensions were prepared with a concentration of 1.6 × 10^6^ conidia per mL, which was considered as a non limiting inoculum for the susceptibility assessment [29]. Cultivar susceptibility was assessed in a separate experiment using adult plants grown in pots. These plants were sown at the same time and inoculated at the same age, as plants from the previously mentioned canopies. For each of the three top leaf ranks, 10 leaves were chosen randomly from fully-developed tillers of 8 plants per cultivar. Leaves were inoculated by applying the spore suspension with a paint brush and then were maintained in a humid atmosphere in order to favor infection [29]. After a latency period, inoculated leaves were removed from plants and scanned. Sporulating areas were delimited manually on scanned pictures and measured using image analysis software ImageJ [30]. The percentage of sporulating area, hereafter called “disease severity”, was used as an indicator of cultivar susceptibility.

### Production of spore fluxes

Simulated rainfall was generated within a rain tower at INRA Thiverval-Grignon [31]. The height of the rain tower (9 m) allowed drops to reach close to terminal velocity before impacting. Two types of rain with drops of calibrated size were generated using the same quantity of water (10 mm = 10L/m^2^) with needles of different sizes. The duration of rain events was of 27 or 47 minutes according to drop size. Drop diameters were measured using a disdrometer (CETP). Drop sizes were chosen to account for diversity of drop diameters commonly encountered in natural rainfalls [32]. Mean drop diameters of ‘light’ and ‘heavy’ rain were 2.09 and 2.47 mm, respectively. Both diameters led to splashing and droplet formation, heavy rain drops have almost twice the kinetic energy of light rain drops [17].

**Fig 2:**
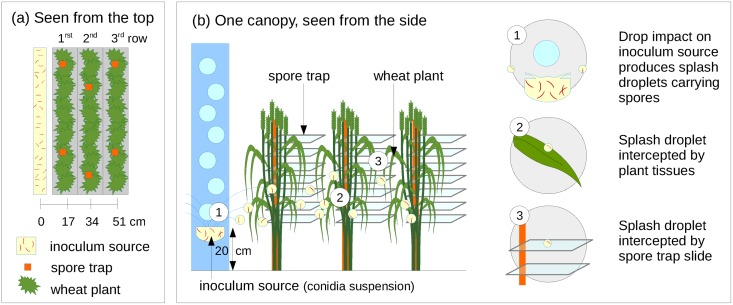
Production of standardized splash droplets and interception by plant canopy. The experimental arrangement is represented here, seen from the top (a) and from the side (b). Calibrated raindrops were generated by a rain simulator and fell on a linear inoculum source constituted of a conidia suspension with a concentration of 1.6 × 10^6^ conidia per mL. Splash droplets traveled through the canopy, some were intercepted by plant tissues or spore trap slides. After a latency period, disease symptoms were observed. They resulted from the number of intercepted spores and from leaf susceptibility. Both spore fluxes and disease symptoms were measured on 10 plants per canopy row.

**Fig 3:**
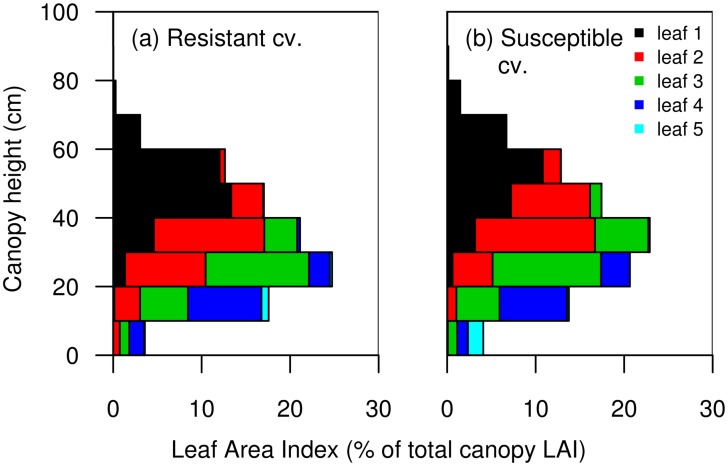
Vertical distribution of leaf area index (leaf area per area of ground). Vertical distribution of LAI was estimated from 3D mock-ups of two wheat cultivars either resistant (a) or susceptible (b) to STB, for each leaf rank. Values were expressed as a proportion of total leaf area index of the canopy. Leaves were numbered from the top to the bottom of the plant. Leaf 1 corresponds to the flag leaf (highest leaf).

Drops were allowed to fall into a shallow spore suspension, which constituted a linear inoculum source with dimensions of 6 x 100 cm, placed beside the first row of each 3-row canopy (Fig 2). The inoculum source was placed at a height of 20 cm, corresponding to the approximate height of the lowest leaves still present on the plant at flowering stage (corresponding usually to leaf rank 4 and lower, Fig 3), which would have constituted the natural source of inoculum in field conditions. The inoculum source was constantly renewed in order to avoid dilution of the conidia suspension (concentration of 1.6 × 10^6^ conidia per mL) by raindrops and to maintain a constant amount of liquid in the linear source [33]. Splash droplets containing spores were thus generated and constituted a repeatable incoming spore flux that was independent of the cultivar susceptibility and canopy architecture. A rain shelter was positioned above the canopy with a window above the inoculum source. This device prevented rain from falling directly on the canopy and avoided subsequent removal of splashed spores or wash-off. Each canopy was inoculated once, by splashed droplets generated during a simulated rain event. For each rain type, two repetitions were carried out for each of the three types of canopy.

### Measurement of spore dispersal within canopies

Horizontal and vertical dispersal of spores resulting from rain splash was measured using spore traps placed within the rows of each canopy, at different distances from the inoculum source. Each trap was composed of 7 microscope slides fixed on a plastic upright, placed at different heights (10 cm between 2 slides) according to canopy height. Two traps were placed within each row, respectively 17, 34 and 51 cm from the inoculum source (Fig 2). Spore trap slides were therefore partially sheltered from splash droplets due to the presence of neighboring leaves of the canopy. Incoming spore flux, *i.e*. not modified by canopy architecture, was characterized by performing the same spore flux measurements, in the same conditions, but without any plants.

Slides were collected after each rain event and photographed using an optical microscope with an automated stage combined with a digital camera as well as the Power Mosaic software (Leica Microsystems, Wetzlar, Germany). Spore density was estimated by averaging visual counts of spores at 20 locations evenly sampled on the slide, for each slide.

### Measurement of plant disease

After each rain event, plants were placed in mist chambers to provide optimal infection conditions [34]. Plants were maintained in a highly humid atmosphere for 3 days following inoculation, thus avoiding any effect of canopy architecture on infection through microclimate. After a latency period, 10 plants (with 2 to 4 tillers each) were collected in each row. Leaves from all plant tillers were removed from plants and disease severity was measured using the same method as for the susceptibility assessment.

In order to fully characterize the relatively small amount of disease symptoms resulting from a single spore dispersal event, several plant disease indicators were assessed. These indicators were measured or computed on a total of 2 391 leaves among which 113 had disease symptoms. Indicators related to the distribution of symptoms included incidence and number of lesions per plant. Incidence was computed, as the proportion of diseased leaves on all tillers of 10 sampled plants per row. In the case of a single event of dispersal, the incidence might not allow one to disentangle differences of dispersal because this indicator does not distinguish if a leaf has been infected more than once by splash droplets. When the susceptibilities of two plants are close (same cultivar), the area of symptoms should be related to the number of intercepted spores. Thus, when plants of the same cultivar in different treatments (pure stand and mixture) were compared, conditional severity and individual lesion size were considered as indicators of the number of spores intercepted at leaf scale. Conditional severity was defined as the mean severity of diseased leaves only (*i.e*., not including healthy leaves). Disease severity was defined as the percentage of sporulating diseased area per leaf. Total lesion area was the sum of all sporulating areas by treatment.

### Data treatment and statistical analysis

Data analysis was performed using the R statistical software [35]. Leaf susceptibility, spore density on spore trap slides and disease severity at leaf scale were not normally distributed according to the Shapiro-Wilk normality test (*P* < 0.05). We therefore used non-parametric tests to assess the impact of each factor (cultivar, leaf rank and rain type) on these three variables. Wilcoxon signed rank tests were used to compare samples two by two. Kruskal-Wallis tests were used to compare more than two samples and to detect shift between distributions.

Multivariate analysis was performed using mean data measured on the first row (2 cultivars in pure stand x 2 rain types x 3 leaf ranks x 2 repetitions = 24 individuals), computed for 3 factors (cultivar, leaf rank, rain type) and for 7 variables (total lesion area at row scale, conditional severity, incidence, leaf susceptibility, leaf surface area and spore fluxes with and without canopy). Total lesion area measured at row scale was used as the variable to be explained in ANOVA. This variable was computed on an average of 22 leaves per leaf rank, for each row. Mean susceptibility was defined as mean disease severity obtained during the susceptibility assessment on 10 leaves per cultivar and leaf rank. Mean spore flux was estimated for each leaf rank as the mean of spore fluxes on slides (2 slides per height, in each row) at the different heights where leaves from the considered leaf rank were present, weighted by the leaf surface area observed at each height. Spore fluxes were also measured without canopy, and included in the analysis as “spore flux unobstructed” in order to account for spore flux gradients perceived by leaves that were most exposed to splash droplets.

Correlation analysis was carried out. Multi-factor analyses of variance (MANOVA) were performed in order to assess the impact of factors on total lesion area. For analysis of covariance (ANCOVA), quantitative covariates (spore flux, leaf surface area, susceptibility) were added individually then together. A simplified model was obtained through model selection using a stepwise algorithm and AIC (Akaike Information Criteria). Model quality was assessed through *R*^2^ values. Interactions were included in the model only when they provided improvements of model quality.

## Results

### Plant characteristics

Susceptibility assessment results confirmed expected differences between the two cultivars (Table 1). Considering all leaf ranks, the susceptible cultivar (Sogood) was on average 3.4 times more affected that the resistant one (Maxwell). Differences between leaf ranks were highly significant (*P* = 3.9 × 10^−6^). The flag leaf (highest leaf) was much more susceptible than lower leaves and the third leaf from the top had almost no disease despite non limiting inoculum and optimal conditions for disease development. Leaf rank was therefore taken into consideration for further analysis.

**Table 1:** Assessment of the susceptibility of two wheat cultivars through leaf severity (% sporulating area) with non limiting inoculum of *Zymoseptoria tritici* conidia and optimal infection conditions.

Leaf rank	Resistant cultivar	Susceptible cultivar	Difference between cultivars (*P*)
1	4.60 (1.65) a	13.85 (3.54) a	0.02
2	1.84 (0.52) a	8.96 (6.52) ab	0.96
3	0.04 (0.04) b	0.91 (0.74) b	0.56

For each leaf rank and cultivar, 10 leaves (sampled from all tillers of 8 plants) were inoculated with a conidia suspension. Plants used for susceptibility assessment were grown in pots and were of the same age and batch as plants used in the splash-dispersal experiments. Mean and standard error (in parenthesis) of severity observed on 10 leaves per leaf rank and per cultivar are presented here. Leaves are numbered from the top of the plant, leaf 1 corresponding to the highest leaf, also called flag leaf. For each line, *P* indicates significance of difference between cultivars, for a given leaf rank. Different lowercase letters indicate significant differences of severity between leaf ranks, for a determined cultivar (Wilcoxon rank sum test, *P* < 0.05).

The resistant cultivar had a larger leaf area and was denser than the susceptible cultivar. Leaf Area Index (LAI) was 4.7 for the resistant cultivar and 3.7 m^2^/m^2^ of ground for the susceptible cultivar. Canopy height (maximum leaf height) was 0.89 m for the resistant cultivar and 0.95 m for the susceptible cultivar. Leaf Area Density (LAD) was 5.3 for the resistant cultivar and 3.9 m^2^/m^3^ for the susceptible cultivar. Distribution of leaf area as a function of canopy height also differed between cultivars (Fig 3). For example, 44% of flag leaf area of the resistant cultivar was located above 50 cm height compared to 63% in the case of the susceptible cultivar.

### Disease distribution

Disease distribution and spore dispersal were assessed through disease symptoms and through spore fluxes. Here, spore fluxes accounted for strictly physical processes as we used a standardized inoculum source which was independent of canopy properties and cultivar resistance. Comparing spore fluxes and disease gradients made it possible to assess the relative importance of physical processes in disease propagation.

**Fig 4:**
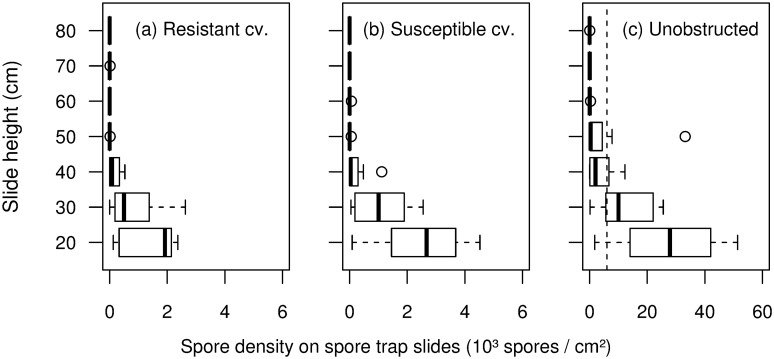
Spore flux as a function of canopy height (spores / cm^2^ of spore trap slide). Spore fluxes were measured 17 cm from the inoculum source for resistant (a) and susceptible (b) cultivar differing by their canopy architecture and in an unobstructed space (c). The vertical dashed line on graph c indicates a spore density of 6 × 10^3^ spores per cm^2^, which is the maximum value for graphs (a) and (b). On each graph, each height corresponds to a total of 8 slides of spore traps (2 slides per canopy x 2 rain types x 2 repetitions). Distributions at 20 and 30 cm height differed significantly between (a) and (b) according to the *χ*^2^ test of independence.

Spore flux depended greatly on vertical (Fig 4) and horizontal (Table 2) distance to the inoculum source. Spore flux decrease with height in the canopy was highly significant (*P* = 6.8 × 10^−14^). Highest spore fluxes were observed up to 10 cm above inoculum source height (20 cm), while hardly any spores were trapped higher than 30 cm above the inoculum source. Most susceptible top leaves therefore received far fewer spores than bottom leaves. In this experiment, we used an artificial source of inoculum. Incoming spore fluxes were therefore similar for both cultivars, for a given rain type. However, spore fluxes measured within the first row differed between cultivars. Spore fluxes trapped within canopies were reduced by up to 10 compared to similar incoming fluxes measured without canopy. In the absence of canopy, many spores were trapped 30 cm above the inoculum source. Comparing spore fluxes with and without canopies (Fig 4), we estimated that the resistant cultivar intercepted 93% of incoming spore fluxes as against only 80% for the susceptible cultivar.

**Table 2:** Disease indicators at different distances from the inoculum source in pure stand and mixture.

Variable	Distance	Susceptible (pure)	Susceptible (mixture)	Resistant (pure)	Resistant (mixture)
Incidence (%)	17 cm	9.23 (2.77)	13.17 (7.52)	9.73 (5.35)	12.62 (4.91)
	34 cm	2.16 (1.28) A	0 (0) B	1.39 (0.81) AB	0.41 (0.41) AB
Lesions per plant	17 cm	4 (1.86)	2.06 (1.49)	1.98 (1.37)	3.75 (1.25)
	34 cm	0.03 (0.03) A	0 (0) B	0.15 (0.1) AB	0.88 (0.54) AB
Conditional severity (%)	17 cm	2.57 (0.78) a	0.55 (0.11) b	0.82 (0.14) ab	1.88 (0.52) a
	34 cm	1.14 (0.29)	0 (0)	0.4 (0.14)	0.38 (0)
Lesion area (cm^2^)	17 cm	0.2 (0.04) a	0.08 (0.01) b	0.14 (0.01) a	0.19 (0.02) c
	34 cm	0.06 (0.01) a	0 (0)	0.11 (0.03) b	0.14 (0) ab

Incidence is defined as the proportion of diseased leaves, computed for each row (10 sampled plants x 4 rows per cultivar and per distance to the inoculum source). Conditional severity is the mean proportion of diseased area of diseased leaves from sampled plants. Lesions per plant is defined as the mean number of lesions observed on each plant of a row. Lesion size is defined as mean size of an individual sporulating lesion. There was no disease on susceptible plants grown in mixture located 34 cm from the inoculum source. Statistical differences between treatments (within a row of the table) according to the Kruskal-Wallis test are indicated by different lowerecase letters for *P* < 0.05 and capital letters for *P* < 0.10. Standard errors are in parenthesis.

Both conditional severity and lesion area of susceptible plants were significantly reduced (*P* < 0.05) in mixture on the first row beside the inoculum source (Table 2). The number of lesions was reduced and the incidence increased for the first row, but this was not significant. On the second row the reduction of the number of lesions and incidence were slightly significant (*P* < 0.10). No disease symptoms were present on susceptible plants of the second row. Disease indicators tended to be higher for resistant plants within mixture compared to pure stand, this was significant for lesion area. Heavy rain caused 2.5 times more diseased area than light rain.

### Relating dispersal patterns to plant characteristics

Lesion area was highly correlated to conditional severity (91%), leaf susceptibility (48%) and incidence (51%). Incidence was correlated to leaf surface area (33%) which played an important role in spore interception. Analysis of variance was used to investigate the relation between total lesion area and factors and quantitative variables (Table 3). Multi-factorial analysis of variance showed that differences of leaf rank and cultivar explained 44% of disease variability. The interaction between cultivar and metamer number was significant (*P* = 2.6 × 10^−3^). However taking into consideration interactions did not improve the model. Analysis of covariance emphasized the importance of leaf surface area. Model including all variables explained 63% of disease variability. The simplified model including only leaf rank and leaf surface area explained 65% of disease variability, which was slightly better than the model including all variables. This suggests that lesion area was strongly related to architectural characteristics of cultivars, here represented by leaf surface area. We therefore emphasize the importance of two factors that contributed to lesion area that are little considered in general: canopy architecture (here leaf surface area) and leaf rank.

**Table 3:** Analysis of variance of disease total lesion area (cm^2^) at row scale resulting from single splash-dispersal events in canopies differing in their architecture and cultivar susceptibility.

	Qualitative variables			Quantitative variables			R^2^
Model	Cultivar	Leaf rank	Rain type	LSA	SFm	LSu	
Qualitative variables only	*	***	NS	-	-	-	0.44
Qualitative + leaf surface area	*	***	NS	***	-	-	0.65
Qualitative + mean spore flux	*	***	NS	-	NS	-	0.46
Qualitative + susceptibility	.	***	NS	-	-	NS	0.43
All variables	*	***	NS	***	NS	NS	0.63
Simplified (AIC)	-	***	NS	***	NS	-	0.65

The analyzed data were the mean variable values (measured 17 cm from the inoculum source) computed at leaf rank scale, corresponding to a total of 24 observations (2 cultivars x 3 leaf ranks x 2 rain types x 2 repetition). The variable to be explained is the sporulating lesion area at leaf rank scale, computed for each row. Quantitative explicative variables include leaf surface area (LSA), mean perceived spore flux (SFm) and leaf susceptibility (LSu). Simplified model was obtained through model selection using a stepwise algorithm and the AIC (Akaike Information Criteria). P codes: 0 < *P* < 0.001 “***”, 0.01 < *P* < 0.05 “*”, 0.05 < *P* < 0.01 “.”, *P* > 0.1 “NS”. Variable not included in the model “-”.

## Discussion

Our results show the contribution of canopy architecture to spore interception and the consequences on disease level resulting from single splash-dispersal events. Wheat leaves are long and different leaf ranks can share similar height in the canopy [36]. These characteristics (Fig 3) were assessed using 3D mock-ups of experimental canopies (Fig 1). We thus quantified the spatial distribution of leaves differing in their susceptibility in relation to spore dispersal gradients.

### Cultivar characteristics

Flag leaf was much more susceptible than other leaf ranks (Table 1). This contrast modulated the vertical disease patterns, as gradients of spore flux and leaf susceptibility were opposed. The difference in susceptibility between leaf layers could be related to differences in leaf senescence, leaf age or leaf rank, as is the case for various pathosystems [37]. *Z. tritici* [16] is often considered as a hemibiotroph fungus as penetration into leaf and primary mycelium development occur in living tissues. An effect of senescence on infection efficiency is therefore consistant and is already used in STB models [38, 39].

The assessment of leaf area distribution outlined notable differences between cultivars which had an impact on their spore interception capacities. The most resistant cultivar was shorter and had a large leaf area which was located close to the inoculum source, compared to the susceptible cultivar (Fig 3). Despite the importance of vertical distribution of leaf area in the case of splash dispersal [40] and its consideration in some models [38, 41], this factor is rarely assessed directly and compared to experimental data. The impact of canopy architecture on dispersal has often been assessed through Leaf Area Index [24], and more rarely through porosity or Leaf Area Density [23]. In field conditions, where inoculum sources are located on older leaves below susceptible leaves, leaf area distribution might also have an impact on vertical spore gradients within the canopy.

### Disease propagation

While the highest quantities of spores were measured close to the inoculum source height (Fig 4), most disease symptoms were observed on the highest and most susceptible leaves. The decrease of spore flux with height is a common splash dispersal pattern [40, 42, 43]. In the case where susceptibility of canopy tissues was relatively homogeneous, this resulted in decreasing disease as a function of canopy height: this was the case in Schoeny *et al*.’s experiment [9]. Our results showed that susceptibility contrast between leaf ranks could strongly modify disease distribution patterns. Smaller spore fluxes (measured with spore traps) were measured within the resistant canopies than in susceptible canopies (Fig 4), this difference was significant 20 cm and 30 cm above the ground. The resistant cultivar had a disease incidence similar to that of the susceptible cultivar (Table 2), despite low susceptibility (Table 1). This might be related to high spore interception, which was consistent with incidence correlation with leaf surface area. Higher barrier effects in cultivar mixtures were associated with larger canopy LAI. These results were in agreement with previous studies showing relations between canopy LAI, spore and disease gradients [9, 20, 21]. A resistant cultivar is expected to provide an efficient barrier effect. It should therefore be dense in order to intercept high quantities of spores. On the other hand, spore interception should be minimized in the case of a susceptible cultivar: leaf area should therefore be lower and the most susceptible leaves should be higher, ie further from the inoculum source.

Conditional severity and lesion size were lower on susceptible plants grown in mixture compared to pure stand (Table 2). Because incoming spore fluxes were independent of the canopy and infectious processes were controlled, we can deduce that on the whole less spores were intercepted by susceptible plants in the mixture than in susceptible pure stand. This can be related to the difference of architecture provided by the resistant cultivar in the mixture. The difference of canopy density did not result in large differences of incidence. This could be explained by the fact that the difference of density did not allow to completely shelter leaves of susceptible plants in pure stand compared to mixture. On other side, the difference of density between treatments led to a significant difference in lesion size on susceptible leaves. Therefore, it made us conclude that the number of droplets carrying spores that reach leaves was reduced with the increase of density but not enough to prevent any spores from reaching a susceptible leaf. Mixed canopies were mainly composed of resistant and dense plants, which probably provided a shelter for susceptible plants within the row. Disease reductions observed in cultivar mixture grown in field conditions (with the same cultivars, in the same proportions) [13] might be partly linked to the interception capacity of the resistant cultivar, related to its large leaf area.

In cultivar mixtures, increasing the proportion of resistant plants is expected to reduce the amount of inoculum. Our conclusions show that, besides effects related to the proportion of each cultivar, the architecture of the canopy can modulate spore transport and interception by leaves.

### Implications for cultivar mixtures design

Airborne disease reduction in cultivar mixtures results mainly from horizontal heterogeneity of plant resistance, obtained by growing plants of different resistance levels in the same field. However, vertical spore transfer is crucial in the case of STB epidemics. Vertical distribution of leaf area index and leaf susceptibility can shape vertical disease gradients resulting from single dispersal events. Different leaf inclinations can lead to different vertical leaf area distribution in wheat canopies, for a similar LAI. This plant characteristic might have an effect on spore interception properties, location of susceptible tissues and horizontal spore transfers. It therefore seems that 3D heterogeneity of cultivar mixtures should be taken into account in order to better understand factors that contribute to splash-dispersed disease reduction in cultivar mixtures. Moreover, these effects might be amplified by several dispersal cycles and contribute to disease reduction in cultivar mixtures. Considering the complexity of disease propagation mechanisms and the high number of possibilities of cultivar mixture designs, spatially explicit modeling could be an interesting way to investigate the impact of cultivar architecture on disease reductions within cultivar mixtures.

The cultivars used in our experiment can easily be grown and harvested together in field conditions. Indeed, they have a similar height and precocity which makes the management of such a mixture close to that of a pure stand. Disease reductions have been obtained in the field with the same cultivars [13, 25]. However, more pronounced differences of architecture between cultivars composing a mixture could be tested. Though some adjustment should be made in crop management, several studies suggest that interesting benefits can be obtained, for example in terms of yield [44, 45] and disease management [46, 47].

## Conclusion

Our results emphasized the importance of the spatial organization of leaves that determines the physical and biological heterogeneity of canopies. Canopy architecture determined interception properties and contributed to disease resulting from single splash dispersal events. Cultivar architecture also contributed to protective properties in a cultivar mixture. This suggests that choosing cultivar architecture according to its function within a mixture could improve mixture efficiency in the case of splash-dispersed diseases. Moreover, we emphasized the importance of vertical canopy heterogeneity in the case of splash-dispersal and suggest that this aspect should be considered, from a biological and physical point of view, in order to understand factors that lead to disease reduction within cultivar mixtures. Taking advantage of the diversity of cultivar architectures might therefore contribute to improving the control of splash-dispersed diseases using cultivar mixtures and to reducing fungicide use.

## Supporting information

S1 Dataset Data presented in figures and tables of the paper. Each worksheet corresponds to a specific Table or Figure of the paper. The correspondence is indicated by worksheet names.
(ODS)
